# Supplementary figures and images for: Apicoplast-derived isoprenoids are essential for biosynthesis of GPI protein anchors, and consequently for egress and invasion in Plasmodium falciparum
Source: PLoS Pathog. 2024 Sep 6;20(9):e1012484. doi: 10.1371/journal.ppat.1012484 (PMC11414934; doi:10.1371/journal.ppat.1012484)

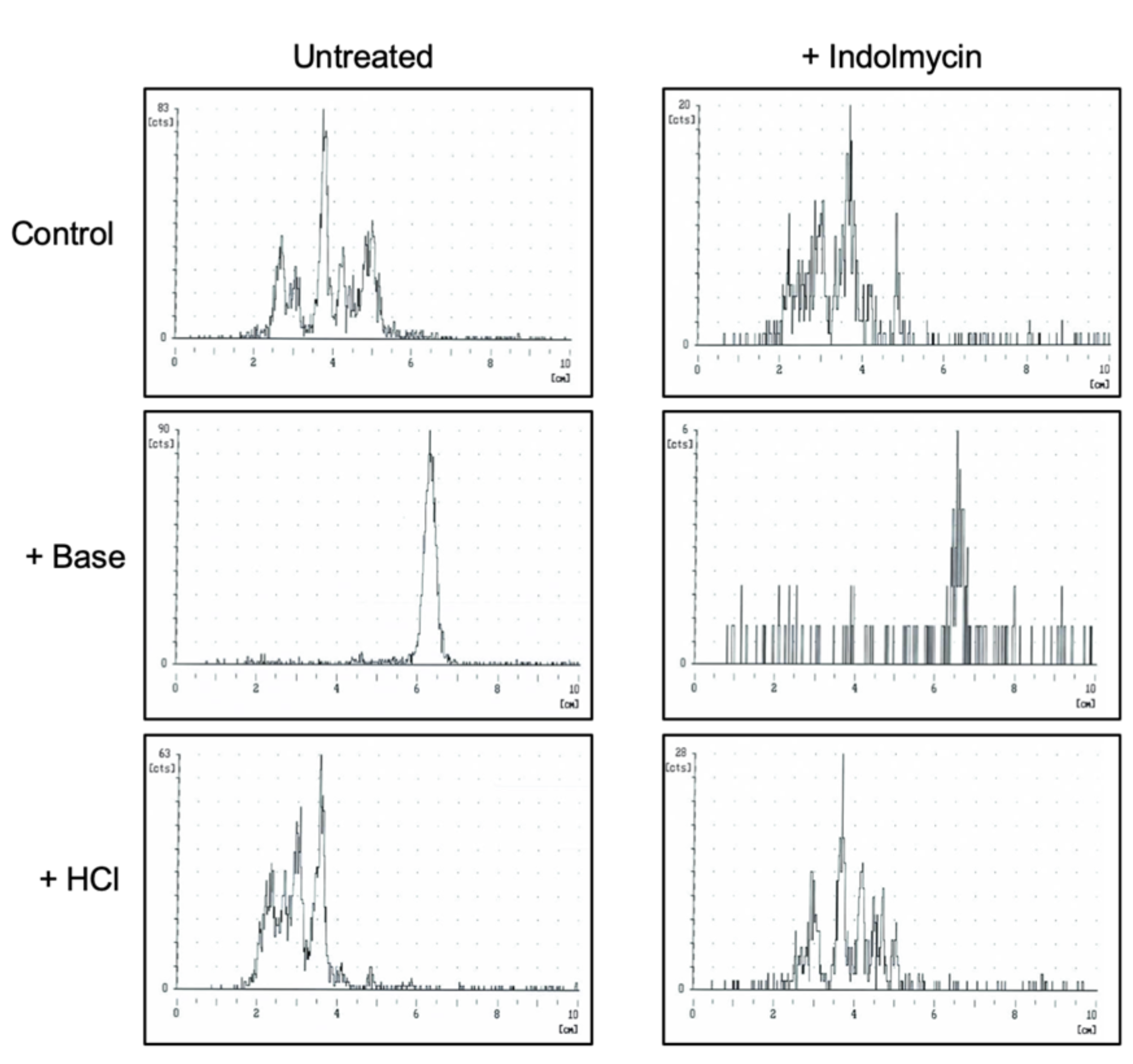

Supplement: S1 Fig — [3H]-labelled glycolipids were resolved by HPTLC before or after mild base or acid treatments (x-axis, cm) and 3H-labelled bands detected by linear ion scanning (y-axis, counts per minute). Note that Dol-P-Man has a faster HPTLC migration after base-treatment due to interference from free fatty acids released from other parasite and red blood cell phospho/neutral lipids. Indolmycin treatment resulted in a 10-fold decrease in 3H-Man incorporation into Dol-P-Man (base-insensitive, acid-sensitive species) and 3-fold decrease in free GPIs (base-sensitive, acid-resistant species). Indolmycin treatment also led to reduced synthesis of mature GPI species (slow migrating species between 2-3cm) indicative of reduced flux into this pathway compared to untreated parasites. (TIFF) [file ppat.1012484.s001.tiff]

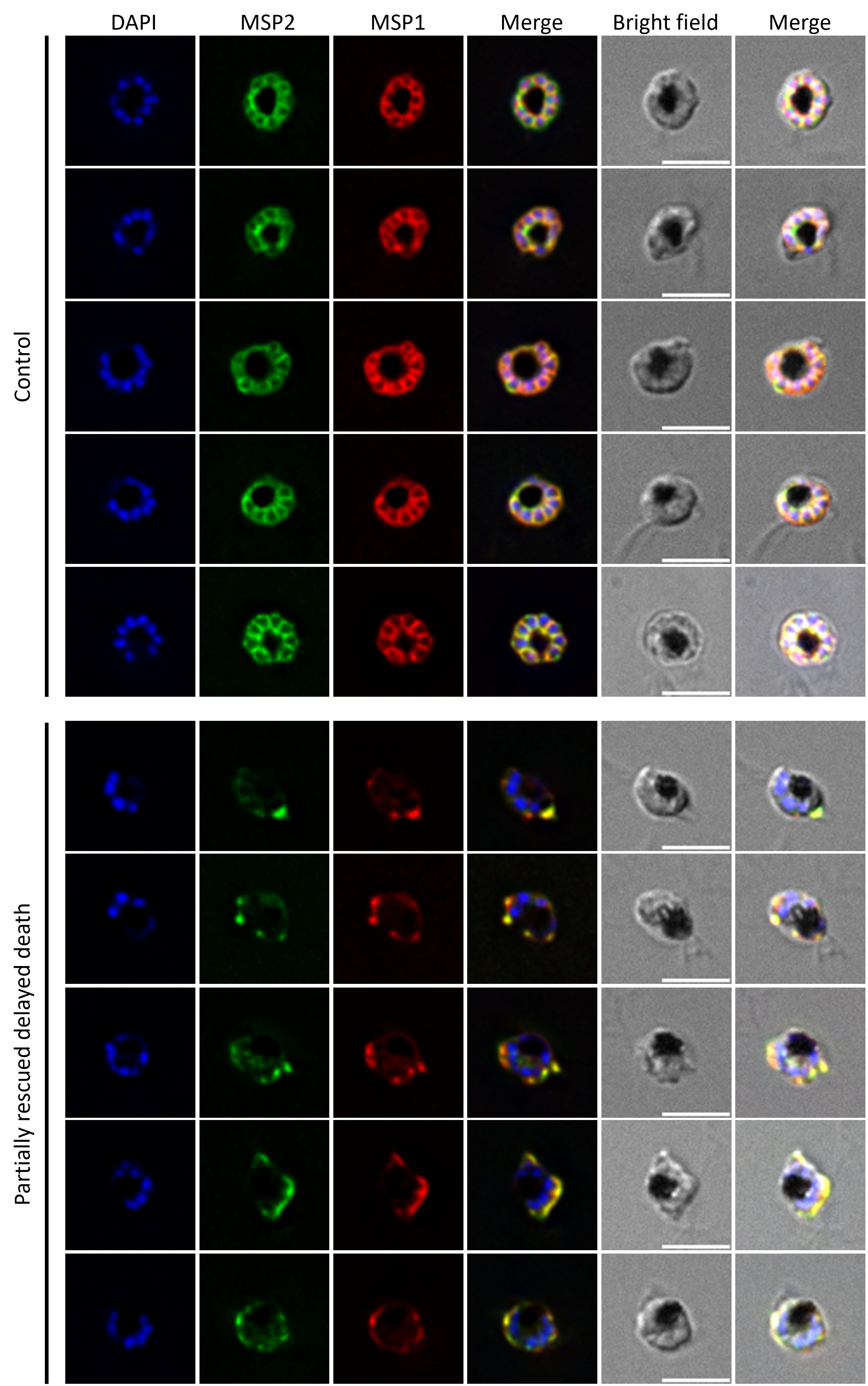

Supplement: S2 Fig — Nuclear visualisation with DAPI stain (blue). Scale bar = 5 μm. (TIFF) [file ppat.1012484.s002.tiff]

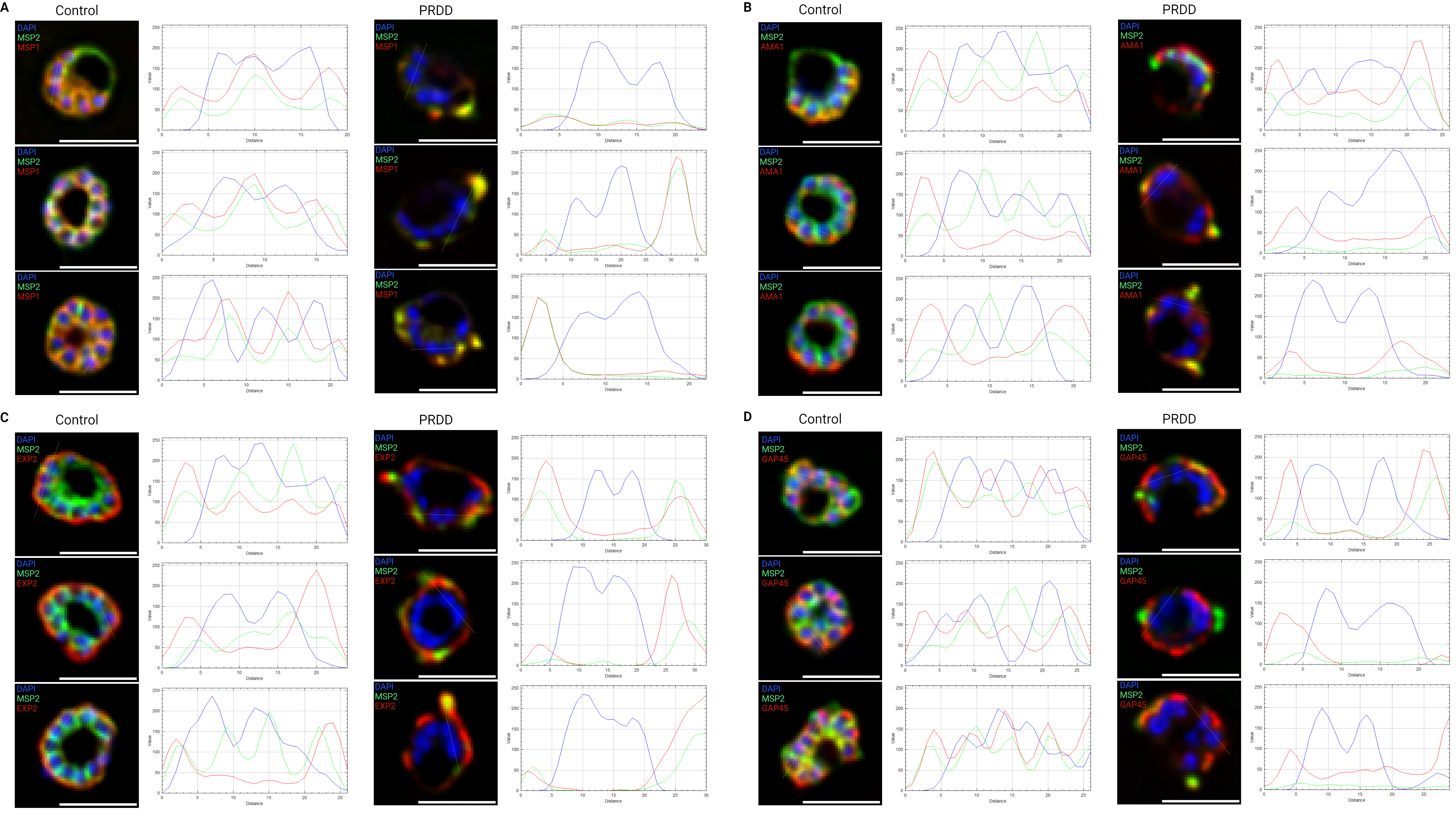

Supplement: S3 Fig — Vectors (yellow) were drawn through 2 to 3 merozoites per schizont. DAPI (blue), MSP2 (green) and A) MSP1, B) AMA1, C) EXP2, D) GAP45 (red). Scale bar = 4 μm. (TIFF) [file ppat.1012484.s003.tiff]

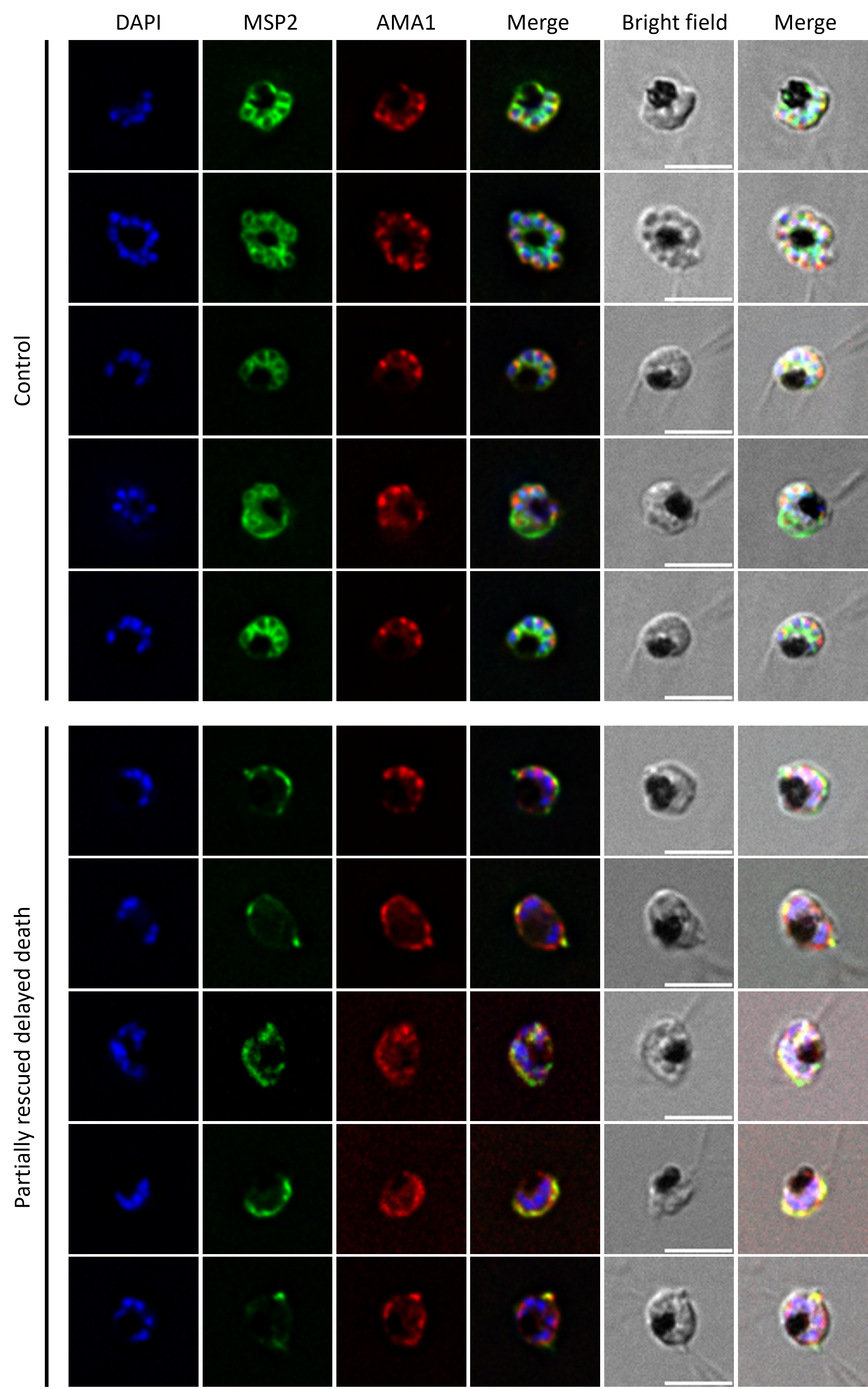

Supplement: S4 Fig — Nuclear visualisation with DAPI stain (blue). Scale bar = 5 μm. (TIFF) [file ppat.1012484.s004.tiff]

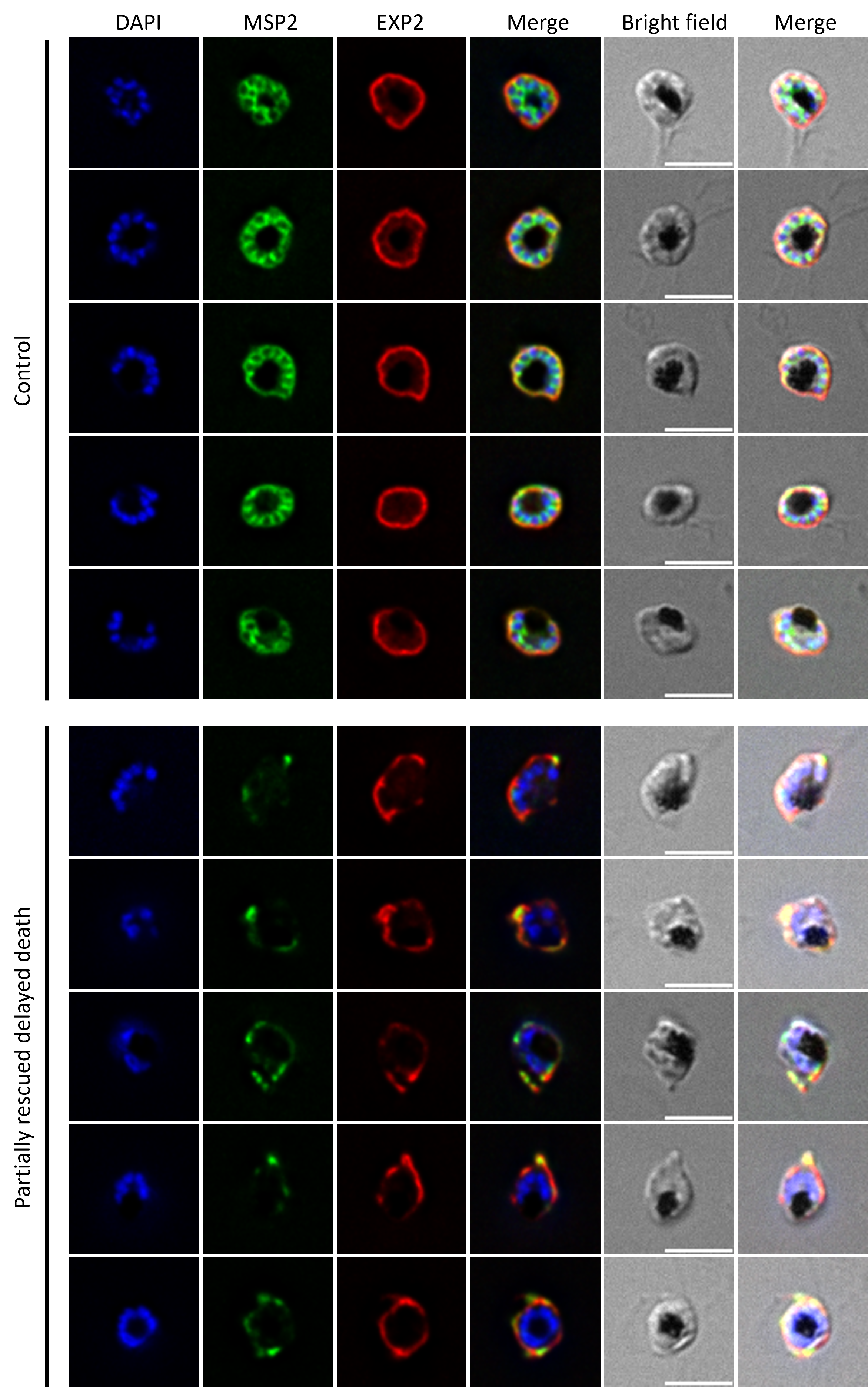

Supplement: S5 Fig — Nuclear visualisation with DAPI stain (blue). Scale bar = 5 μm. (TIFF) [file ppat.1012484.s005.tiff]

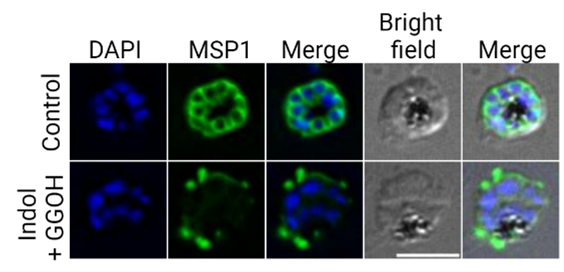

Supplement: S6 Fig — Treatment with apicoplast translation inhibitor indolmycin (Indol) with GGOH results in the mislocalisation of surface GPI-anchored protein merozoite surface protein 1 (MSP1). Nuclei stained with 4,6-diamidino-2-phenylindole (DAPI; blue). Scale bar = 5 μm. (TIFF) [file ppat.1012484.s006.tiff]

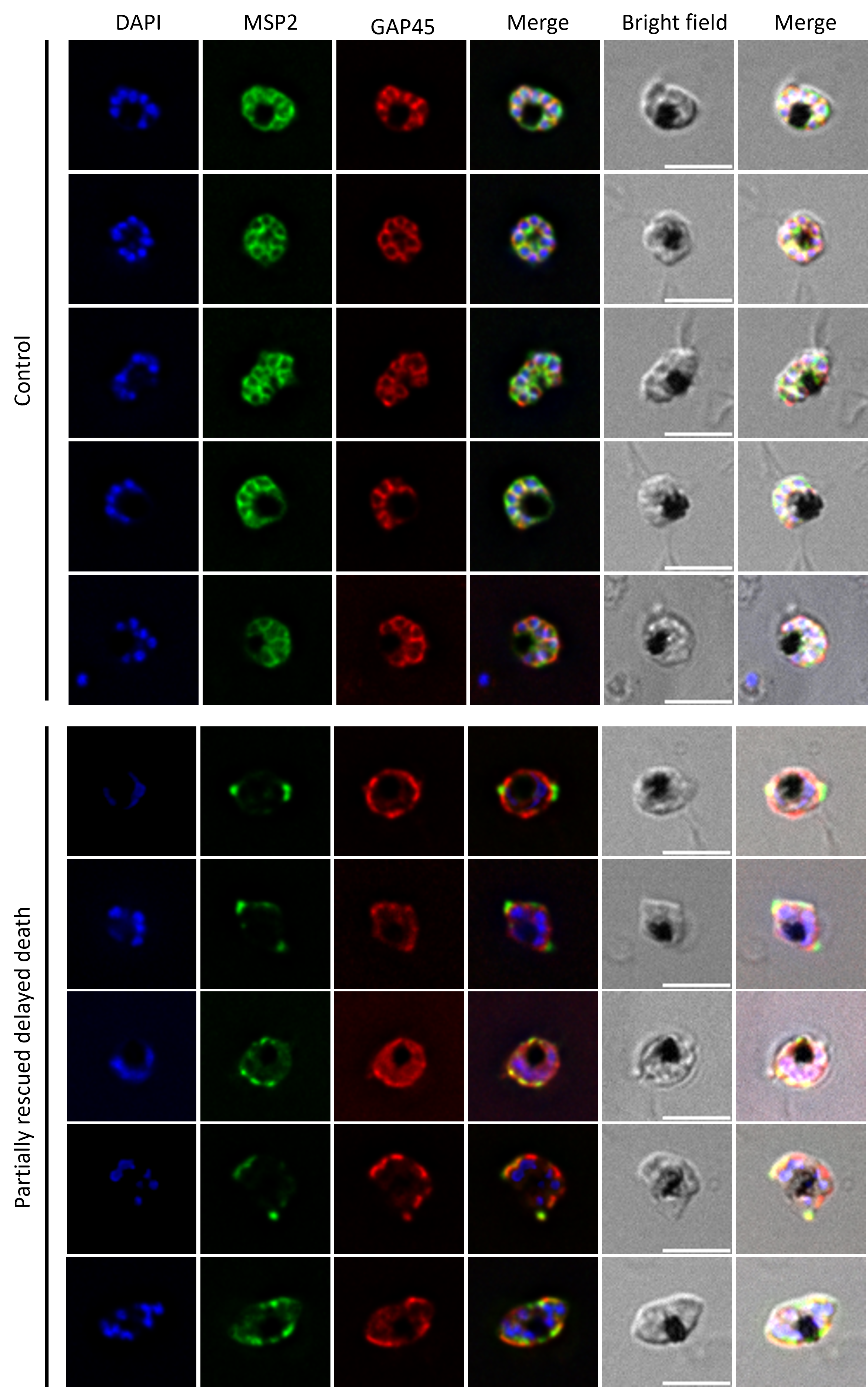

Supplement: S7 Fig — Nuclear visualisation with DAPI stain (blue). Scale bar = 5 μm. (TIFF) [file ppat.1012484.s007.tiff]

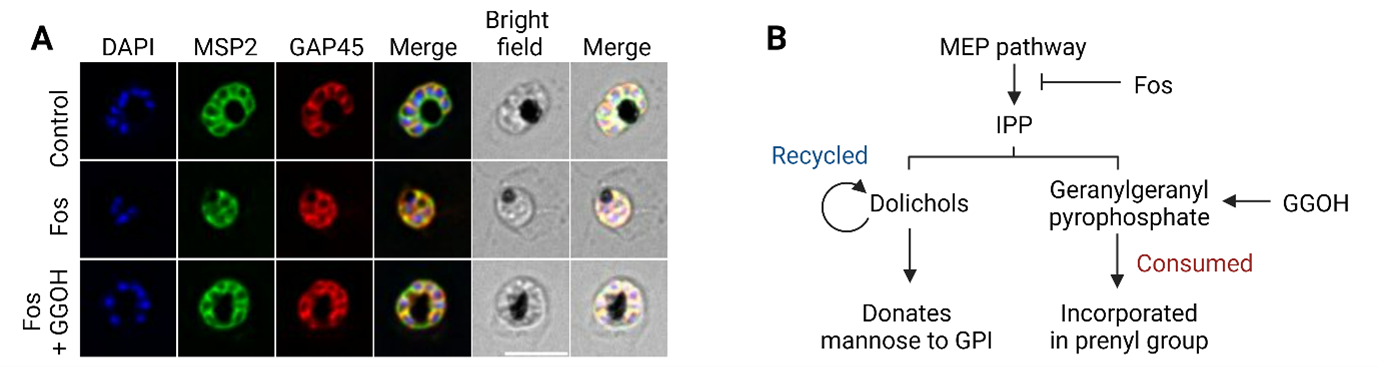

Supplement: S8 Fig — A) Fosmidomycin (Fos) treatment is lethal, killing at the trophozoite stage. Addition of geranylgeraniol (GGOH) allows parasites to progress to the schizont stage where GPI-anchored MSP2 (green) maintains surface localisation. Nuclei stained with DAPI (blue). Images represent single Z stacks. Scale bar = 5 μm. B) Dolichol and geranylgeranyl pyrophosphate are both synthesised from apicoplast-derived IPP. Dolichols are recycled in their role as sugar donor and so are more robust to IPP inhibition. Geranylgeranyl pyrophosphate is consumed to form the prenyl group itself and following fosmidomycin treatment is quickly depleted with lethal consequences. (TIFF) [file ppat.1012484.s008.tiff]

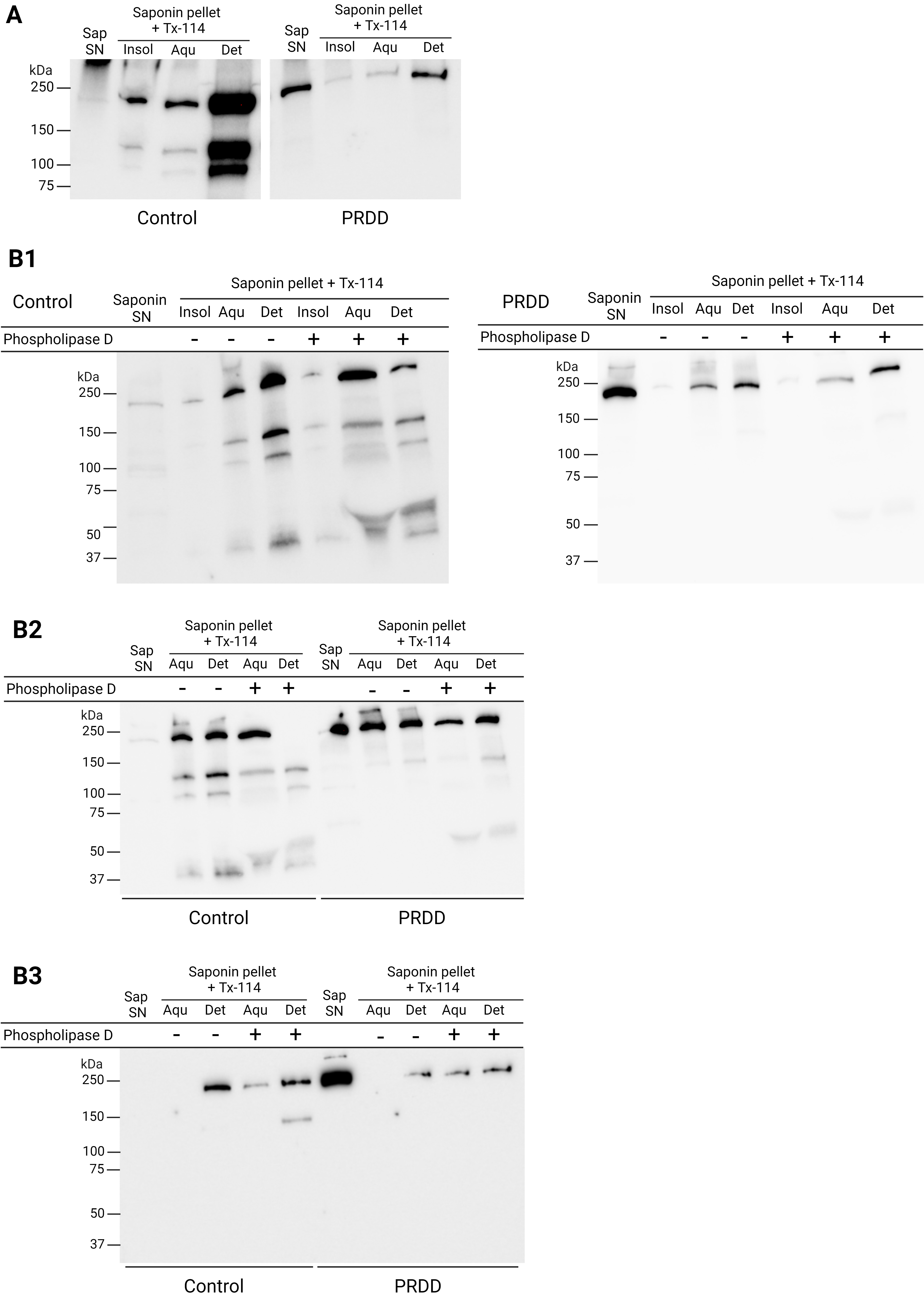

Supplement: S9 Fig — A) RBCs infected with schizont stage P. falciparum were first lysed with low concentrations of saponin (0.03% w/v) to collect proteins soluble in the RBC and parasitophorous vacuole (Sap SN). The remaining parasite material was then incubated with TX-114 for phase partitioning into detergent fraction (Det) containing membrane associated proteins and an aqueous (Aqu) fraction with soluble proteins. Proteins insoluble to TX-114 were also loaded (Insol). B) Biological replicates show that the GPI anchor can be cleaved with phospholipase C, resulting in proteins typically associating with membranes becoming untethered. (TIFF) [file ppat.1012484.s009.tiff]

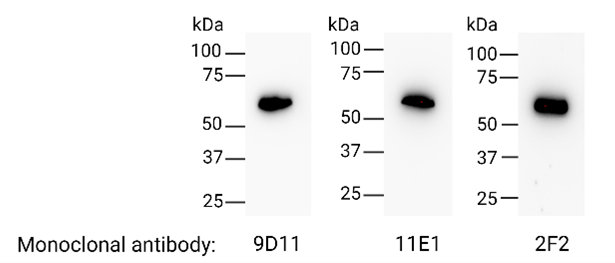

Supplement: S10 Fig — Western blot of 3D7 parasite lysates probed with anti-MSP2 monoclonal antibodies show MSP2 consistently migrating at a size larger than the predicted 28 kDa. (TIFF) [file ppat.1012484.s010.tiff]

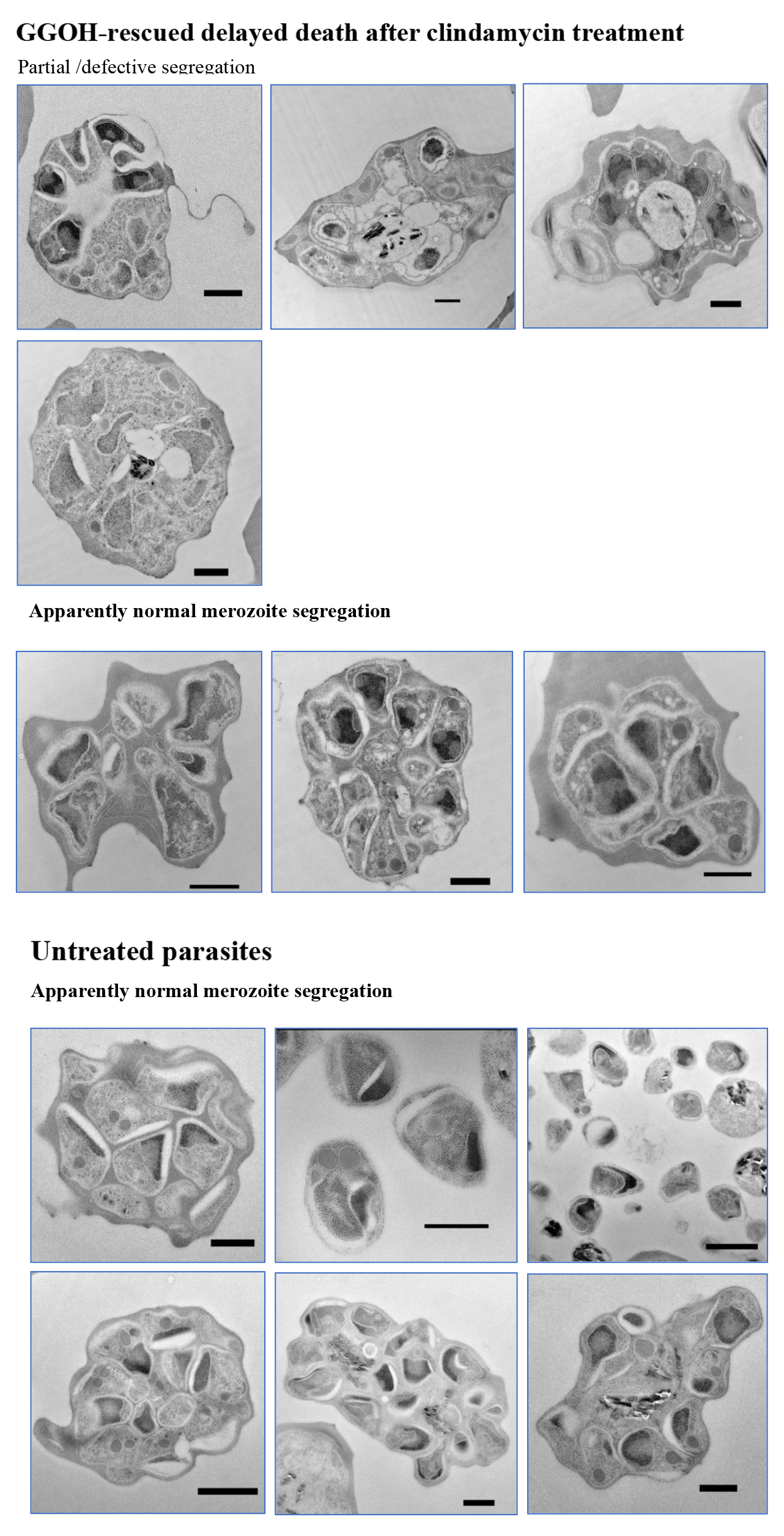

Supplement: S11 Fig — Schizonts in untreated conditions possess clear and total segmentation of nuclei, rhoptry formation and membrane encapsulated merozoites, whereas most parasites with partially rescued delayed death have abnormal segmentation with incomplete creating of membranes around new nuclei. Some parasites in PRDD also have apparently morphologically normal segmentation. All scale bars are 1μm. (TIF) [file ppat.1012484.s011.tif]
